# Dental pulp-derived stromal cells exhibit a higher osteogenic potency than bone marrow-derived stromal cells in vitro and in a porcine critical-size bone defect model

**DOI:** 10.1051/sicotj/2016004

**Published:** 2016-04-20

**Authors:** Jonas Jensen, Claus Tvedesøe, Jan Hendrik Duedal Rölfing, Casper Bindzus Foldager, Helle Lysdahl, David Christian Evar Kraft, Muwan Chen, Jorgen Baas, Dang Quang Svend Le, Cody Eric Bünger

**Affiliations:** 1 Orthopaedic Research Laboratory, Aarhus University Hospital Noerrebrogade 44 8000 Aarhus C Denmark; 2 Department of Orthodontics, School of Dentistry, Aarhus University Vennelyst Boulevard 9 8000 Aarhus C Denmark; 3 Interdisciplinary Nanoscience Center (iNANO), Aarhus University Gustav Wieds Vej 14 8000 Aarhus C Denmark; 4 Department of Orthopaedics, Aarhus University Hospital Noerrebrogade 44 8000 Aarhus C Denmark; 5 Department of Radiology, Aarhus University Hospital Noerrebrogade 44 8000 Aarhus C Denmark

**Keywords:** Animal Model, Dental Pulp, Tissue Engineering, Scaffolds, Bone Regeneration

## Abstract

*Introduction*: The osteogenic differentiation of bone marrow-derived mesenchymal stromal cells (BMSCs) was compared with that of dental pulp-derived stromal cells (DPSCs) in vitro and in a pig calvaria critical-size bone defect model.

*Methods*: BMSCs and DPSCs were extracted from the tibia bone marrow and the molar teeth of each pig, respectively. BMSCs and DPSCs were cultured in monolayer and on a three-dimensional (3D) polycaprolactone (PCL) – hyaluronic acid – tricalcium phosphate (HT-PCL) scaffold. Population doubling (PD), alkaline phosphatase (ALP) activity, and calcium deposition were measured in monolayer. In the 3D culture ALP activity, DNA content, and calcium deposition were evaluated. Six non-penetrating critical-size defects were made in each calvarium of 14 pigs. Three paired sub-studies were conducted: (1) empty defects vs. HT-PCL scaffolds; (2) PCL scaffolds vs. HT-PCL scaffolds; and (3) autologous BMSCs on HT-PCL scaffolds vs. autologous DPSCs on HT-PCL scaffolds. The observation time was five weeks. Bone volume fractions (BV/TV) were assessed with micro-computed tomography (μCT) and histomorphometry.

*Results and discussion*: The results from the in vitro study revealed a higher ALP activity and calcium deposition of the DPSC cultures compared with BMSC cultures. Significantly more bone was present in the HT-PCL group than in both the pure PCL scaffold group and the empty defect group in vivo. DPSCs generated more bone than BMSCs when seeded on HT-PCL. In conclusion, DPSCs exhibited a higher osteogenic potential compared with BMSCs both in vitro and in vivo, making it a potential cell source for future bone tissue engineering.

## Introduction

Functionalization of scaffolds for bone tissue engineering is a key factor for promoting new bone formation. Most bulk materials for scaffold construction lack sufficient osteoinductive and osteogenic properties to ensure a satisfactory stimulation of bone healing [1]. One approach to functionalize scaffolds includes the seeding of autologous bone marrow-derived stromal cells (BMSCs) onto the scaffolds prior to implantation. BMSCs are known to initiate new bone formation by differentiating into osteoblasts, by stimulating the bone healing environment, and by recruiting stem cells as well as osteoblasts from the adjacent tissue [2]. Stimulating bone formation using BMSCs has been promising in both animal studies and human trials [3, 4]. However, due to donor site morbidity, low cell number upon harvest, and loss of phenotypic behavior during culturing, other potent sources of osteogenic stem cells have been investigated [5]. Dental pulp stromal cells (DPSCs) originating from the dentin/pulp complex represent an accessible cell source that can be isolated easily and contain a subset of dental pulp stem cells [6]. Dental pulp stem cells are known to express bone-related markers similar to BMSCs and seem to be able to produce more colony forming units, survive for a longer time, and elicit a higher proliferation rate than BMSCs [7, 8]. Dental pulp stem cells have displayed the ability to differentiate into osteoblasts and produce both mineralized and extracellular matrix as well as a bone-like trabecular structure [9–11].

Polycaprolactone (PCL) is one of the most widely used synthetic polymers in scaffold development and application within orthopedics. The polymer is highly biocompatible and degrades into non-toxic waste products metabolized by beta-oxidation. Fused deposition modeling (FDM) is a rapid prototyping technology, which ensures that each scaffold can be custom-made to fit perfectly into different bone void dimensions. PCL scaffolds manufactured by FDM have previously demonstrated promising potential as a backbone for further functional modifications [11–13]. Coating the polymer surface with other bioactive compounds such as hyaluronic acid (HA) can be used to increase the scaffold surface area. HA is a naturally occurring non-immunogenic glycosaminoglycan and plays a significant role as a facilitator of osteogenic differentiation and as a migration stimulating agent for BMSCs [14]. By adding β-tricalcium phosphate (β-TCP) to the coating solution, a well-known osteoconductive source can be added to the scaffold surface. This HA/β-TCP coating has shown favorable osteogenic properties in a recent in vitro and subcutaneous mouse study [15].

Our main aim was to compare DPSC and BMSC cultures with regard to proliferation, osteogenic differentiation, and mineralization in vitro and afterwards bone formation in a large-animal critical-size defect. Further aims were to investigate whether HA/β-TCP modifications to PCL scaffolds enhanced bone healing compared with either an empty defect or a pure PCL scaffold.

## Materials and methods

### Scaffold fabrication

The scaffolds were made of polycaprolactone (PCL, Perstorp, UK) polymers with a molecular weight of 50 kDa. Using a BioScaffolder (Sys + Eng GmbH, Germany), the scaffolds were built from layered deposition of polymer strands by extruding molten PCL from an extrusion die with an inner diameter of 200 mm (final fiber diameter ~ 175 mm). To increase surface hydrophilicity, the scaffolds were treated with NaOH as described previously [11]. These scaffolds are hereafter referred to as *PCL scaffolds*. PCL coating with HA and β-TCP is performed as previously described [11]. The final coated HA-TCP PCL scaffolds are referred to as *HT-PCL scaffolds*.

The scaffolds were characterized using scanning electron microscopy (SEM) (Nova NanoSEM 600, FEI Company, Eindhoven, the Netherlands) and micro-computed tomography (μCT) (μCT 40, Scanco Medical AG, Zürich, Switzerland).

### Study design, animal model

**Figure 1:**
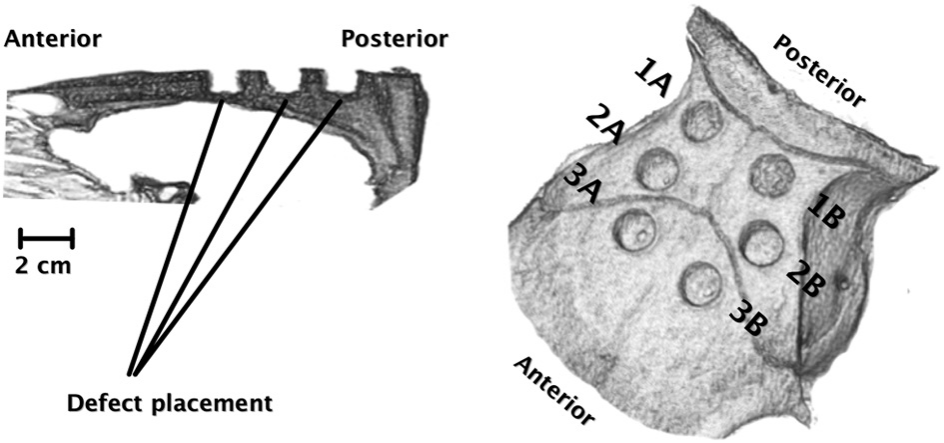
CT reconstructions one week after surgery (0.6 mm thick, non-overlapping sections, 60 mA, 120 kV) acquired using a SOMATOM Definition CT scanner (Siemens AG Medical Solutions, Erlangen, Germany) and visualized using OsiriX v.5.7.1 64-bit (Bernex, Switzerland). The left reconstruction illustrates a sagittal view of the position and depth of the drill holes in relation to the cranial cavity. Note that the calvaria thickness decreases toward the anterior part. On the right, the three sub-studies, 1A/1B, 2A/2B, and 3A/3B, are illustrated in relation to the cranial sutures.

Fourteen, skeletally mature, female Danish Landrace pigs (age: 537 ± 19 days, weight at surgery: 190 ± 32 kg) were used. The calvarium was chosen because of the trabecular morphology of the bone as well as the large uniform area ideal for multiple group assessment. Three paired comparisons were chosen on the basis of a relatively high variability in bone healing from the posterior to the anterior part of the calvaria [12]: posterior (1), in the middle (2), and in the anterior (3) part of the calvaria as illustrated on the CT reconstruction in Figure 1. The two groups in each sub-study were randomly distributed in relation to the sagittal axis as follows:

(1A) Empty defect vs. (1B) HT-PCL scaffold; (2A) PCL scaffold vs. (2B) HT-PCL scaffold; (3A) HT-PCL scaffold with BMSCs, vs. (3B) HT-PCL scaffold with DPSCs (Figure 1). *N* = 14 for each group.

The study was approved by the Animal Experiments Inspectorate, Denmark and conformed to Danish law (Application No. 2012-15-2934-00362). Medication prior to surgery and cell extraction procedures were performed according to a previous study [12].

### Cell extraction and cultivation

One month prior to surgery, mononucleated cells from premolar teeth and bone marrow were extracted from each individual pig. Using a dental elevator, a premolar from the upper and lower jaw was loosened from the surrounding ligaments and extracted with an extraction forceps. The pulp from the premolar teeth was retrieved and incubated at 37 °C for 30 min in Dulbecco’s Modified Eagle’s Medium (DMEM) (Gibco, 31966, Life Technologies Europe BV, Naerum, Denmark) containing 3 mg/mL of collagenase type I (Worthington Biochemical Corporation, Freehold, NJ, USA) and 2.4 units/mL of dispase II (Roche Diagnostics, Mannheim, Germany). Pulp-derived stromal cells liberated from the pulp were passed through a 70 μm strainer (BD Biosciences-Discovery Labware, Bedford, MA, USA) [8, 10]. Hereafter, only cells from one of the premolar teeth extracted from each individual pig will be used to conduct the following studies.

Cells were expanded in DMEM, supplemented with 10% fetal bovine serum (FBS) (Gibco, 10270) [*proliferation media*] and antibiotics (25,000 IU/mL of penicillin and 25 mg/mL of streptomycin; DuraScan Medical Products, Odense, Denmark) at 37 °C.

Bone marrow (30 mL) was aspirated from the proximal tibia using a 50 mL syringe containing 10 mL DMEM with 50 IE heparin (LEO Pharma A/S, Ballerup, Denmark). The amount of bone marrow aspirated was kept at a clinically relevant quantity while still sufficient to acquire enough BMSCs for further studies. Mononucleated cells from the bone marrow aspirate were isolated by Ficoll^®^ gradient centrifugation (Sigma-Aldrich Co. LLC., St. Louis, MO, USA) and expanded in proliferation media. The medium was changed twice a week. Upon near confluence, DPSCs and BMSCs were detached using 0.25% trypsin (Gibco) and 0.1% EDTA (Invitrogen, Taastrup, Denmark) in phosphate-buffered saline (PBS). Cells were expanded by serially passaging and underwent two passages before seeding onto HT-PCL scaffolds.

Five days prior to surgery, 2.5 × 10^6^ DPSCs or BMSCs from each pig were seeded on HT-PCL scaffolds in agarose-coated wells and allowed to expand for two days in proliferation media. Afterwards, the media were changed to osteogenic media, which comprised of 10 nM dexamethasone (D2915, Sigma-Aldrich), 10 mM β-glycerophosphate (G9422, Sigma-Aldrich), and 283 μM L-ascorbic acid 2-phosphate (A8960, Sigma-Aldrich) added to the proliferation media [*osteogenic media*]. Osteogenic differentiation was stimulated for three days in vitro before implantation.

### In vitro osteogenesis in monolayer

Osteogenic potential of DPSCs and BMSCs was assessed from cell cultures extracted from three randomly chosen pigs (DPSC #1-3 and BMSC #1-3). The cells were seeded in 175 cm^2^ flasks (Almeco, Esbjerg, Denmark) with a density of 4000 cells/cm^2^ and expanded in proliferation media. Following expansion, a baseline was set for the monolayer studies. To evaluate proliferation, the two cell types were cultured separately in proliferation media for up to 40 days. Cells were counted using a Bürker Türk counting chamber to calculate population doublings (PDs). Media were changed twice a week.

The ALP activity of both cell types was determined by a microspectrophotometric end-point assay after four and seven days. Absorbance of p-nitrophenol was measured at 405/650 nm using a Victor^3^ microspectrophotometer microplate reader (PerkinElmer Life Sciences, Waltham, MA, USA).

Calcium deposition was assessed with alizarin red (AZR) staining after 14 and 21 days. Cells were fixed in chilled 70% ethanol for 60 min followed by rinsing in double-distilled water (ddH_2_O) before staining with a 0.2% AZR (A5533, Sigma-Aldrich) ethanol solution for 15 min on a tilting table. Non-specific staining was minimized by five ddH_2_O rinses. The plates were subsequently dried for 24 h. Red stain of calcium mineralization was observed by phase contrast microscopy. After quantitative de-staining using 200 μL of 5% SDS in 0.5 M HCl, the AZR concentration (ng/mL) was estimated at wavelengths of 405/600 nm using a microspectrophotometer microplate reader, Victor 3 1420 Multilabel Counter (PerkinElmer Life Sciences).

### In vitro osteogenesis on scaffolds

To evaluate cell proliferation and osteogenic differentiation on HT-PCL scaffolds, 12 scaffolds (Ø = 10 mm, *h* = 10 mm) for each of the six individual cell cultures were placed in agarose-coated wells and seeded with 1.1 × 10^6^ cells. Scaffolds were cultured in proliferation media for two days followed by culture in osteogenic media. On day 5 (equal to T_0_ implantation in the in vivo experiment), day 12 (T_7_), and day 19 (T_14_), four scaffolds from each cell culture were analyzed for DNA quantification, ALP activity, and calcium deposition using protocols previously described [11].

Monolayer culture studies were conducted with six technical replicates and scaffold culture studies with four replicates for each cell line.

### Surgical procedure, follow-up and termination

Six non-penetrating defects were created in the pig calvaria using a custom-made flattened cannulated drill bit (Ø = 15 mm, *h* = 10 mm). The surgical procedure and postoperative care regimen have been described previously [12]. After five weeks, a lethal dose of pentobarbital was injected. Subsequently, the frontal and parietal bone was resected en bloc and frozen. Afterwards, rectangular blocks encompassing each drill defect were cut and fixed in chilled 70% ethanol prior to further processing.

### μCT and histomorphometry analysis

Specimen preparation, μCT, and histomorphometry were performed similar to previous studies [12, 16]. Briefly, specimens were embedded in cold methylmethacrylate and scanned using high-resolution μCT scanning (μCT 40, Scanco Medical AG, Zürich, Switzerland). Afterwards, a random vertical cut through the center axis of the cylinder was made. The first slice for histomorphometry was chosen at random within 100 μm of the center surface and slices with a thickness of 7 μm (increment 10 μm) encompassing the entire defect and adjacent bone were cut on a microtome. Histomorphometry was performed by two independent blinded observers using an Olympus microscope (Olympus, Ballerup, Denmark) with Visiopharm Integrator System software (newCAST, v. 3.4.1.0, Visiopharm A/S, Horsholm, Denmark). Intra- and inter-observer variance was determined and point count technique was utilized to quantify BV/TV.

### Statistics

Results followed a normal distribution when analyzed using D’Agostino’s K^2^ test. The in vitro difference was calculated from grouped means (BMSC and DPSC). Data in figures are presented as mean ± *SD*. Mean values were compared using two-way ANOVA with repeated measures (time × cell type). Sidak’s test was used to correct for multiple comparisons. The three paired in vivo sub-study comparisons were calculated as group mean with standard deviations and compared using Student’s *t*-test. A *p*-value of less than 0.05 was considered statistically significant. STATA software (version 12.1 StataCorp LP, College Station, TX, USA) and PRISM (version 6, GraphPad Software Inc., San Diego, CA, USA) were utilized for all calculations.

## Results

### In vitro monolayer and 3D culture

**Figure 2.**
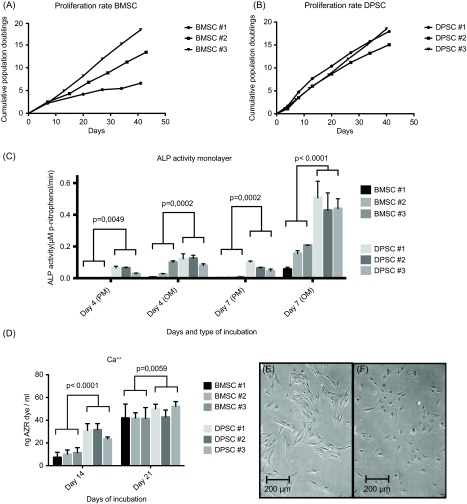
In vitro monolayer culture study of BMSCs and DPSCs. The accumulated population doublings of BMSC #1-3 (a) and DPSC #1-3 (b) are depicted as points on graph with connecting lines. The graphs illustrate the diversion in PD rate in between the three BMSCs compared with the uniform PD curves of the DPSCs. The ALP activity (c) and concentration of Ca^++^ (d) analyses are presented as mean histograms of each individual cell line with error bars representing the technical standard deviations. *P*-values are based on a combined group mean for each cell type. Microscope images of BMSCs (e) and DPSCs (f) are represented from day one in the monolayer experiment setup. The morphological difference between the two cell lines is illustrated.

BMSC#1-3 expressed similar rate of PDs during the first two weeks of culture. After two weeks, PDs of BMSC#1-3 showed large variance while PDs of DPSC#1-3 increased more homogeneously (Figures 2a and 2b). DPSC ALP activity was significantly higher in both types of culture media at each time point compared with BMSC (Figure 2c). The difference was most pronounced in proliferation media. After 14 days of culture in osteogenic medium Ca^2+^ deposition was two times higher for DPSC #1-3 compared with BMSC #1-3. The difference in Ca^2+^ was less pronounced after 21 days although still significantly higher for DPSCs (*p* = 0.006). Cell morphology during proliferation prior to baseline of the monolayer study revealed that BMSCs were large and spindle-shaped whereas the DPSCs were small and round (Figures 2eand 2f).

**Figure 3.**
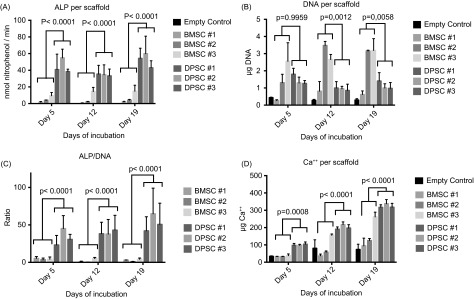
In vitro HT-PCL scaffold seeded with BMSCs or DPSCs. The group mean of the ALP, DNA, and Ca^++^ assays as well as ALP/DNA ratio are presented as mean histograms. Error bars represent technical standard deviations and *p*-values were calculated on combined cell type means. ALP, DNA, and Ca^++^ assays were supplemented with a negative empty HT-PCL scaffold control (*n* = 4) at all time points.

The two cell sources expressed different behavior when cultured on HT-PCL scaffolds with BMSC #1-3 mainly proliferating and DPSC #1-3 undergoing osteogenic differentiation. Already on day 5, DPSC #1-3 elicited a significantly higher ALP activity, which was maintained throughout the experiment (Figure 3a). BMSC #1-3 showed virtually no ALP activity while expressing a significantly higher quantity of DNA detected after 12 and 19 days (Figure 3b). The pronounced difference in the ALP/DNA is depicted in Figure 3c. The osteogenic differentiation of DPSC #1-3 resulted in a significantly higher Ca^2+^ deposition at all time points compared with the BMSC #1-3 (Figure 3d).

### Micro-computed tomography (μCT)

**Figure 4.**
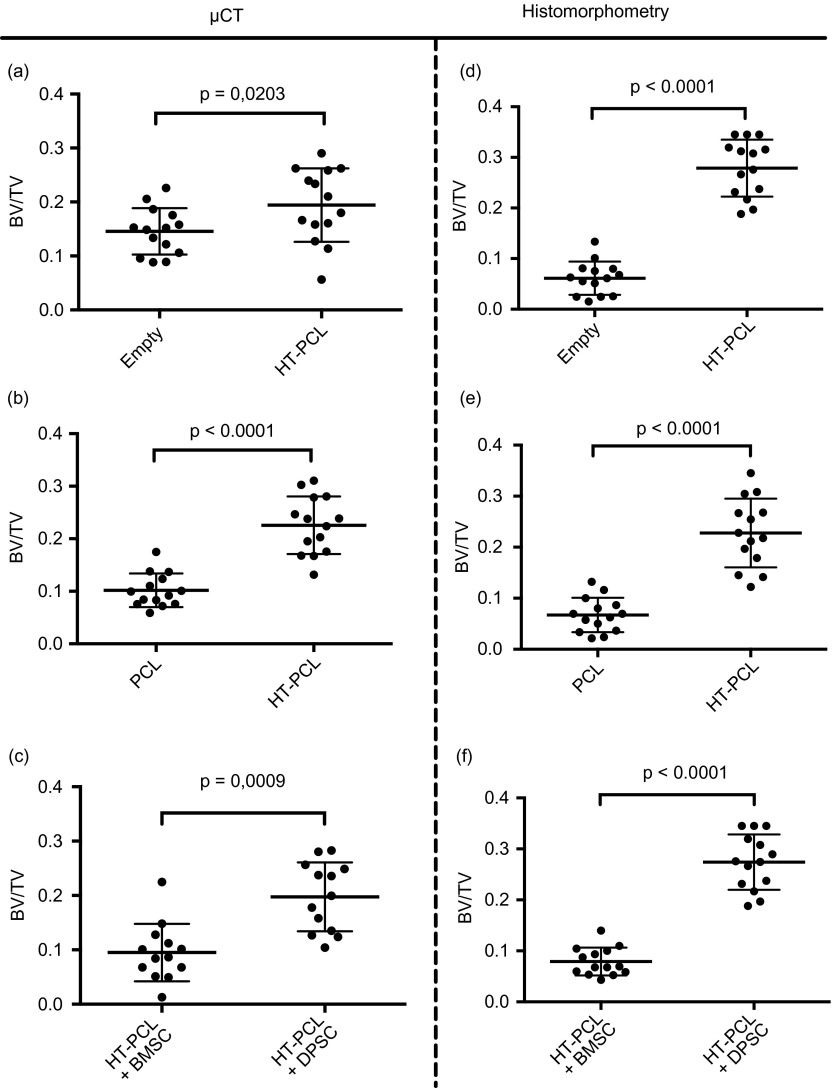
In vivo BV/TV results estimated with μCT and histomorphometry are presented as scatter plots. Individual data points are depicted with dots and middle bar represents the mean. Top and bottom bar represents standard deviations. The top two scatter plots (a and d) represent the paired sub-study with an empty defect and a HT-PCL scaffold. Middle two scatter plots (b and e) illustrate the PCL vs. HT-PCL sub-study, whereas bottom scatter plots (c and f) represent autologous BMSCs on HT-PCL scaffolds vs. autologous DPSCs on HT-PCL scaffolds.

In vivo sub-study 1 compared the healing of an empty defect with a defect with a HT-PCL scaffold and revealed a significantly higher BV/TV when the scaffold was utilized (mean^diff^ = 0.0486, *SD*^diff^ = 0.0688) (Figure 4a). It was evident looking at the three-dimensional reconstructions that bone was regenerated throughout the defect in contrast to the empty defect where new bone formation was restricted to the edges. In sub-study 2, a significantly higher BV/TV was observed in the HT-PCL group compared with the pure PCL scaffold (mean^diff^ = 0.1239, *SD*^diff^ = 0.0688) (Figure 4b). In sub-study 3 comparing BMSCs or DPSCs seeded onto HT-PCL scaffolds a significantly higher BV/TV was observed in the DPSC group (mean^diff^ = 0.1025, *SD*^diff^ = 0.0846) (Figure 4c).

### Histomorphometry

Histomorphometry was comparable with the μCT results. BV/TV in sub-study 1 was significantly higher in the HT-PCL group than in the empty defects (mean^diff^ = 0.2175, *SD*^diff^ = 0.0796) (Figure 4d). In sub-study 2 the HT-PCL group showed a significant higher BV/TV compared with the PCL group (mean^diff^ = 0.1607, *SD*^diff^ = 0.0709) (Figure 4e). In sub-study 3, a higher BV/TV was observed in the DPSC group than in the μCT results (mean^diff^ = 0.1951, *SD*^diff^ = 0.0594) (Figure 4f).

**Figure 5.**
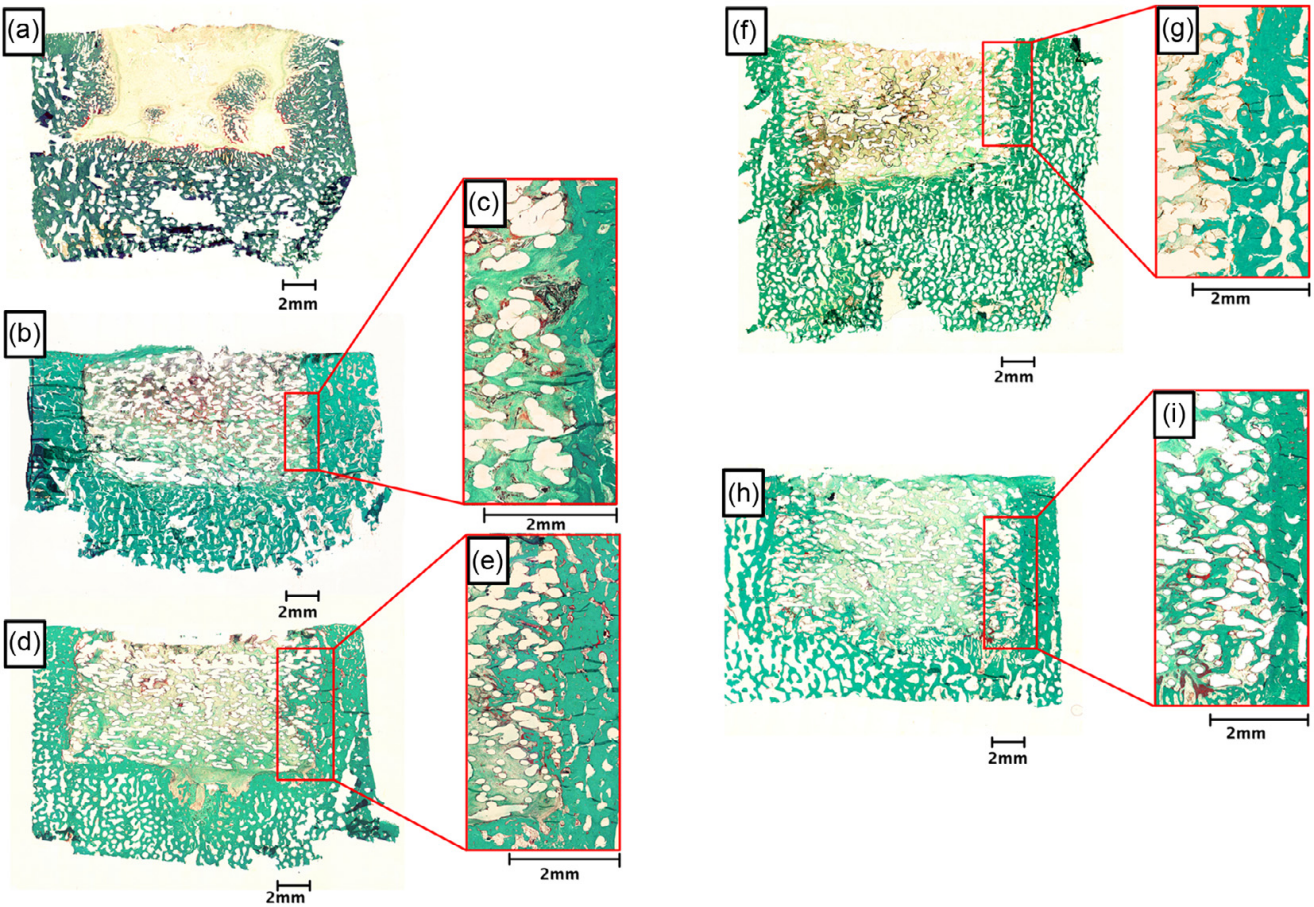
Representative images of histological sections stained with Goldner’s Trichrome. The histological sections depict: (a) empty defect or defects treated with (b) PCL scaffold; (d) HT-CPL scaffold; (f) BMSCs on HT-PCL scaffolds; and (h) DPSCs on HT-PCL scaffolds. Superimposed magnifications of the transition area between defect and existing bone are presented for each of the scaffold-containing groups. The magnifications are: (c) PCL scaffold; (e) HT-CPL scaffold; (g) BMSCs on HT-PCL scaffolds, and (i) DPSCs on HT-PCL scaffolds. Translucent areas within the defects treated with a PCL scaffold represent areas where PCL was present prior to embedding. Scale bars are presented at each image.

In the empty defect group, only small ossification areas and sparse bone ingrowth from the adjacent bone was observed (Figure 5a). In defects treated with PCL scaffolds alone (Figure 5b), little new bone formation was observed, and the gaps between the PCL fibers were filled with fibrous tissue. Almost no bone ingrowth was observed from the edges, and osteoid was sparsely present (Figure 5c). Treatment with the HT-PCL scaffold (Figure 5d) resulted in an increased bone ingrowth from the walls of the defect compared with the PCL group. Furthermore, osteoid was more prevalent in the defects, but the center part of the scaffold remained primarily filled with fibrous tissue and scaffold material. The HT-PCL scaffolds seeded with BMSCs before implantation showed similar bone ingrowth morphology to the pure HT-PCL scaffold (Figure 5g). In many of the specimens where DPSCs were seeded before implantation, small islands of bone tissue were present also in the center of the defect (Figure 5h). Furthermore, the ingrowth from the walls of the defect was more pronounced (Figure 5i). No morphological differences in the bone structure were observed between the groups.

## Discussion

The most notable finding in the present study was the increased regenerative osteogenic potential of DPSCs compared with BMSCs, which to the authors’ knowledge has not previously been shown in a large-animal model. Here, we show that the osteogenic effect of DPSCs was significantly higher than the effect of BMSCs both in vitro and in vivo. Furthermore, the HT-PCL scaffold showed good osteoconductive properties compared with both an empty control and a PCL scaffold without surface modification. This corroborates favorable osteoconductive properties from the surface coating previously documented by our research group [11]. Although the bulk PCL component exhibits some mechanical strength, the deficiency in the compressive modulus when compared with native bone tissue excludes this and similar scaffolds from use without additional support at load-bearing defect sites.

While BMSCs seeded on different carriers and scaffolds for bone defect repair in animals have been investigated by several other groups, dental pulp stem cells have primarily been investigated as a facilitator for osseointegration of metal implants [17, 18]. We observed that osteogenic differentiation was evident at an earlier time point leading to more pronounced mineralization using DPSCs compared with BMSCs *in vitro*. ALP and AZR assays from monolayer culture illustrated a slower and less pronounced osteogenic differentiation and mineralization of BMSCs. This effect may partially be explained by the previously described decrease in proliferation and osteogenic potential of BMSCs with increasing PD [19]. The PD rate of DPSCs was slightly higher than BMSCs, but perhaps more importantly, the PD rate of DPSC cultures was more homogeneous than the PD rate of BMSC cultures. Furthermore, it has previously been suggested that BMSCs are comprised of a heterogeneous mixture of cells in various stages of differentiation and that the proportions of non-osteogenic, osteoprogenitors, and committed osteogenic cells could vary significantly among donors [20]. This could be another reason for the observed difference in the BMSC proliferation rate. However, since we applied a commonly used method for BMSC harvest, these heterogeneity issues might be expected to apply to the general use of BMSCs.

Calcium deposition showed a more uniform and larger increase within the DPSC cultures, whereas the BMSC cultures were more irregular. A reason for this observation could be the initial state of the two different types of cell cultures. Dental tissues are specialized and do not undergo remodeling to the same extent as in bony tissue [21]. DPSCs may therefore be more committed to osteogenic differentiation already at day one, almost resembling the behavior of a pre-osteoblast. Since DPSCs would include a population of cells other than dental pulp stem cells, a sub-population of cells present in the culture already committed to an osteogenic/odontogenic lineage would be likely. This assessment is emphasized by the presence of ALP activity in DPSC monolayer seeded in proliferation medium after only four days. Dental pulp stem cells have previously been shown to be able to differentiate into dentin-forming odontoblasts [7]. In most studies however, culturing has caused a differentiation toward the osteogenic lineage capable of forming bone [8, 10, 22]. Further in vitro and in vivo characterization of the stromal cell cultures could possibly have clarified some of the differences between the osteogenic potential of DPSCs and BMSCs. In similar comparative studies, analysis of surface antigen expression as well as ectopic bone formation and immunohistochemical staining was performed in order to further characterize the cell cultures [23]. However, this was not within the scope of this study.

Because of the bone morphology in the calvaria and interference of the scaffold compound, we were unable to state with certainty whether trabecular bone alone was formed in the defects with DPSCs or if some dentin-like matrix was present. However, no morphological difference was observed between the bone formed in the DPSC and BMSC defect group. Because of the advantageous attributes of DPSCs over BMSCs seen in this study, therapeutical application of DPSCs in large bony defect healing or improvement of implant fixation could become relevant for oral and maxillofacial surgeons as well as orthopedic surgeons. Akin to extraction of BMSCs, obtaining DPSCs from either extracted wisdom teeth or from pulpectomy can be expected to cause patient morbidity. However, a high proportion of the population already undergo wisdom teeth extraction creating a readily available, high yield, cell source for cryopreservation and future utilization [24, 25].

The female landrace pig was the animal of choice owing to its large calvaria surface area and depth as well as healing rates comparable to humans (1.2–1.5 mm/day vs. 1.0–1.5 mm/day) [26]. The pigs were 18 months at surgery, making the specimens skeletally mature and thereby ensuring the feasibility to create critical-size defects within the defined parameters [12, 27]. Calvaria bone healing is theoretically mediated via intramembranous ossification rather than endochondral ossification. However, endochondral ossification has also been observed in calvarial bone healing [28]. In conclusion, this study showed superior bone healing potential of DPSCs compared with BMSCs both in vitro and in a large-animal bone defect. The cause of this finding may partially be explained by a faster and more homogeneous DPSC differentiation into osteogenic lineage. The HA and β-TCP coating of the PCL scaffold increased new bone formation compared with a pure PCL scaffold and an empty defect.

## Conflict of interest

JJ, CT, JHDR, ABF, HL, DCEK, MC, JB, DQSL, and CEB declare no conflict of interest.
